# HPV shapes tumor transcriptome by globally modifying the pool of RNA binding protein-binding motif

**DOI:** 10.18632/aging.101927

**Published:** 2019-04-29

**Authors:** Yingcheng Wu, Hao Chen, Yuyan Chen, Lishuai Qu, Erhao Zhang, Zhou Wang, Yuanyuan Wu, Riyun Yang, Renfang Mao, Cuihua Lu, Yihui Fan

**Affiliations:** 1 Laboratory of Medical Science, School of Medicine, Nantong University, Jiangsu 226001, China; 2 Department of Gastroenterology, Affiliated Hospital of Nantong University, Jiangsu 226001, China; 3 Department of Surgery, Affiliated Hospital of Nantong University, Jiangsu 226001, China; 4 School of Life Sciences, Nantong University, Jiangsu 226001, China; 5 Department of Pathophysiology, School of Medicine, Nantong University, Jiangsu 226001, China; 6 Department of Immunology, School of Medicine, Nantong University, Jiangsu 226001, China; * Equal contribution

**Keywords:** oncogenic viruses, alternative polyadenylation, RNA-binding protein, RBM25, PD-1, aging, age-related disease

## Abstract

Human papillomavirus (HPV) positive head and neck cancer displayed specific transcription landscape but the underlying molecular mechanisms are not fully determined. Here, we interestingly found that HPV infection could globally elongate the 3’-untranslated regions (3’UTRs) in the majority of alternative polyadenylation (APA)-containing genes. Counterintuitively, the 3’UTR elongation does not affect their resident gene expression. Rather, they significantly increase the number of binding sites for RNA-binding proteins (RBPs) and subsequently upregulate a group of oncogenic genes by absorbing RBPs. A significant fraction of HPV affected genes are regulated through such mechanism that is 3’UTR-mediated recruitment of RBPs. As an example, we observed that HPV infection increases the length of 3’UTR of RBM25 transcript and hence recruits much more RNA binding protein including FUS and DGCR8. Consequently, in the absence of FUS and DGCR8 regulation, PD-1 was rescued and up-regulated after HPV infection. Taken together, our findings not only suggest a novel paradigm of how oncogenic viruses shape tumor transcriptome by modifying the 3’UTR, but also present a previously unrecognized layer of APA—RBP interplay in this molecular hierarchy. Modification of the pool of RBP-binding motif might expand our understandings into virus-associated carcinogenesis.

## Introduction

Cancer is a group of diseases involving abnormal and aggressive cell growth accompanied by loss of tissue-specific function. It has already become one of the most challenging diseases that affect health globally, but its etiology is still not fully understood. Several known risk factors such as viral infection, tobacco use, obesity, and excessive drinking of alcohol can significantly increase the incidence of a subtype of cancer [1–4]. In particular, the oncogenic viruses are thought to cause ~20% of all human cancers [5]. Yet, the underlying mechanism of how viruses induce cancers remains poorly defined.

In general, there are seven known human oncogenic viruses, including human papillomavirus (HPV), hepatitis B virus (HBV), hepatitis C virus (HCV), human T- lymphotropic virus 1 (HTLV-1), Epstein–Barr virus (EBV), Kaposi sarcoma- associated herpesvirus (KSHV), and Merkel cell polyomavirus (MCPyV) [6]. HPV is a small DNA virus and infects mucosal epithelia [7]. Its infection is frequently associated with cervical carcinoma as well as head and neck squamous carcinomas (HNSCs) [8,9]. Throughout the chronic infection period, oncogenic viruses using oncoproteins to shape cellular processes for replication, immune evasion, and cellular transformation. Oncoproteins directly interact and modify the function of key cellular proteins such as tumor suppressors (pRB and p53), PI3K–AKT–mTOR signaling, MAPK signaling and NF-κB signaling [10–13]. High-throughput sequencing studies revealed that HPV induced profound transcriptional alterations in HNSCs [14]. It suggests that besides direct modification of protein function, oncoproteins also profoundly shape transcriptome. However, the underlying mechanisms about how virus shape cellular transcriptome are not fully defined.

Alternative polyadenylation (APA) was shown to regulate the inclusion of sequences into the 3’-untranslated regions (3’UTRs) and thus involved in multiple biological processes by affecting the stability, localization, and half-life of the mRNAs [15]. Evidence from pan-cancer analysis revealed that a majority of APA-associated genes show shorter 3’UTR compared with normal samples [16]. The shortened 3’UTRs make their resident genes resistance to be attacked by miRNAs [17,18], which validates the link between 3’UTR and gene expression and perfectly explains how proto-oncogenes were activated. We hence hypothesized that alternative 3’UTR length will also possibly change the binding sites for RNA-binding proteins (RBPs) and thus disrupt the RNA output in the post-transcriptional level. Due to the critical role of APA in the mRNA output, we questioned whether oncogenic virus such as HPV could control the APA process to achieve immune evasion and cellular transformation.

To date, freshly published computational tools and datasets [16] make it possible to explore those unanswered questions. Here, by using high-throughput profiling analyses over large patient cohorts, we interestingly found that HPV infection could globally elongate the 3’UTRs in the majority of APA-containing genes. Counterintuitively, the 3’UTR elongation does not affect their resident gene expression, but strikingly, recruit RBPs and reshape cellular transcriptome for cell proliferation and immune evasion.

## RESULTS

### HPV infection globally elongates 3’UTRs

It remains an open question of how HPV impacts host cells and induces oncogenic changes. Recent findings have suggested the biological significance of miRNAs in HPV positive tumors [19,20], where miRNAs bind to 3’UTRs of key molecules such as IL-1α. These discoveries support the importance of 3’UTR and hence raise the question of whether HPV infection affects 3’UTR directly.

**Figure 1:**
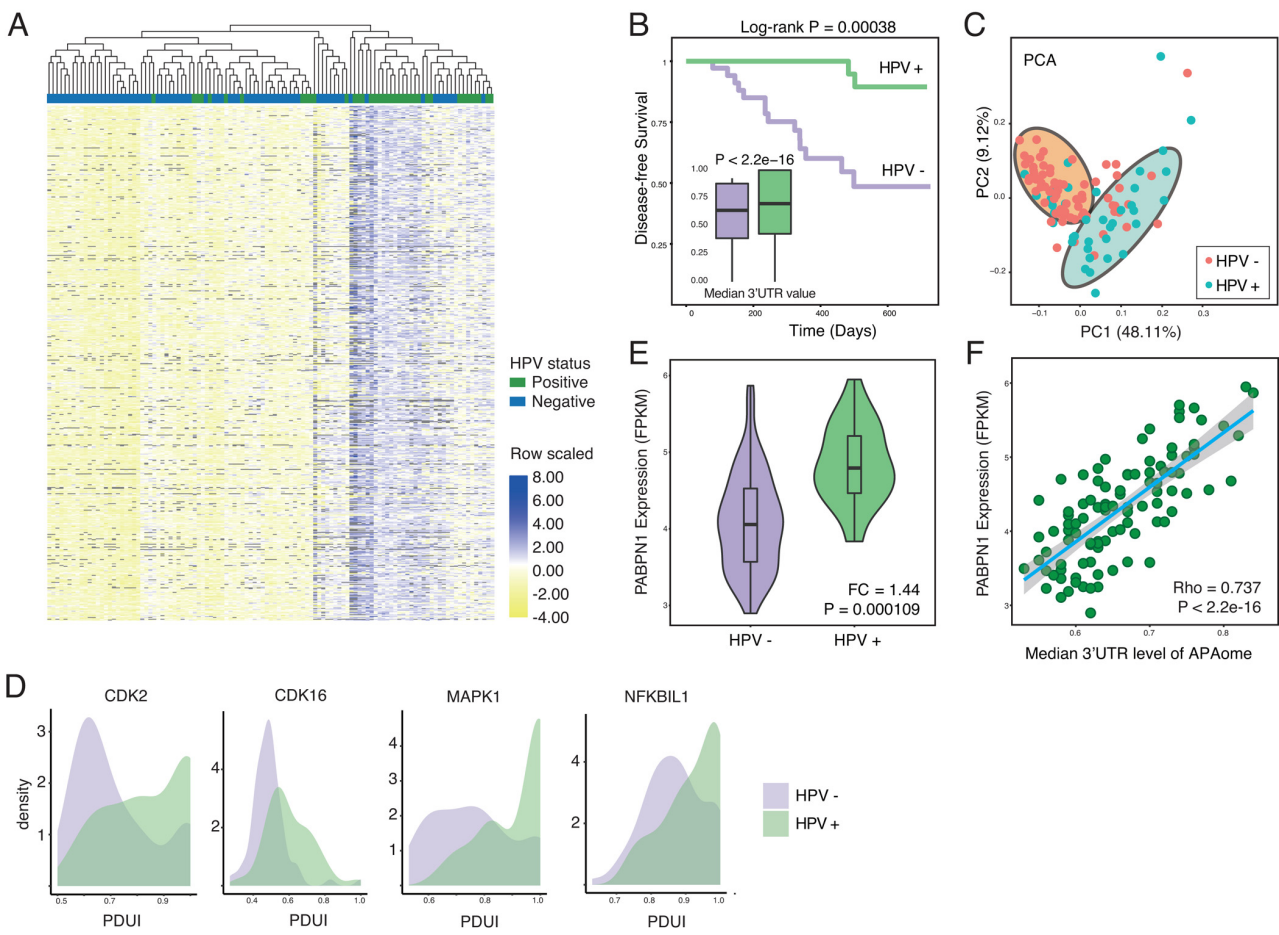
**HPV infection causes globally longer 3’UTR in HNSC.** (**A**) Among all APA events, 54.53% of genes show significant longer 3’UTR length (1855 of 3402, Wilcoxon test, excluding terms with computation uncertainties). HPV+ samples harbor significant longer 3’UTR. The heat map shows the top 100 APA events ranked by P-value (Wilcoxon test). Values are scaled by rows. (**B**) HPV infection status associates the prognosis of HNSC patients. In general, all APA events shows universally longer 3’UTR in HPV+ HNSC samples compared to HPV- samples in the level of APAome. (**C**) The principle component analysis of all head and neck cancer patients based on all APA events, showing the different HPV infection status. Each dot corresponds to each patient sample, colored according to HPV infection status. (**D**) Key hallmark cancer genes show different 3’UTR profiles between HPV+ and HPV- samples. (**E**) The length of 3’UTR can be used to distinguish the status of HPV infection. Principle component analysis was conducted based on all APA events excluding null values. PC1 and PC2 were showed. (**F**) PABPN1, the master regulator of APA, is significantly upregulated in HPV+ samples. Fold change and P-value were computed through EdgeR.

To test our hypothesis, we first compute the PDUI for APAome in both HPV+ and HPV- HNSC samples. PDUI is a widely-accepted quantifying unit for evaluating APA level by using RNA-seq data (Methods and Supplementary Table 1) [16,21]. After generating the PDUI profiles, we perform the differential APA analysis between HPV positive and negative samples (Methods). Strikingly, among the whole APAome within 8831 events, 4824 events show differential APA. With a close look, we find that significant 3’UTR lengthening (3’UL) events predominate (1855 of 3402 longer 3’UTR (54.53%), 1492 of 3402 unchanged 3’UTR (43.86%), 55 of 3402 shorter 3’UTR (1.62%) cut-off |logFC| was set as 0.2). The top 100 differential APA events are shown in Figure 1A. Among the APAome, HPV mediates the globally longer 3’UTR length (Figure 1A and 1B). Next, we conduct the principal component analysis and found that 3’UTR length differentiates the HPV positive and negative tumors clearly (Figure 1C). The 3’UTR of several hallmark genes such as Cyclin-dependent kinase 2 (CDK2), Cyclin-dependent kinase 16 (CDK16), MAPK1 and NFKBIL1 are significantly longer in HPV positive cancer compared with HPV negative cancer (Figure 1D). Together, our results clearly demonstrate that HPV infection could globally increase the length of 3’UTR.

The APA process is orchestrated by multi-protein machinery consisting of a list of key components [21]. To understand the underlying molecular mechanism, we further analyze the expression of individual key components [21] in HPV positive and negative cancers (Supplementary Figure 1A and Figure 1E). Interestingly, almost all essential APA regulators show the trend of upregulation. In particular, PABPN1 shows significant higher expression in HPV positive cancers (Figure 1E). The previous report reveals that PABPN1 is a master regulator which governs the APA processes [22], where higher expression of PABPN1 marks the suppression of alternative cleavage and polyadenylation [21]. Consistent with the known function of PABPN1, the upregulation of PABPN1 in HPV infected cells might explain the universal lengthening of 3’UTR. Further analysis clearly indicates a positive correlation between PABPN1 level and 3’UTR lengthening (Figure 1F), while other components do not show a similar trend as PABPN1 (Supplementary Figure 1B). In summary, HPV infection profoundly affects the length of 3’UTR probably through upregulation of PABPN1, the key component of 3′-end processing machinery.

### 3’UTR lengthening does not affect their resident gene expression

**Figure 2:**
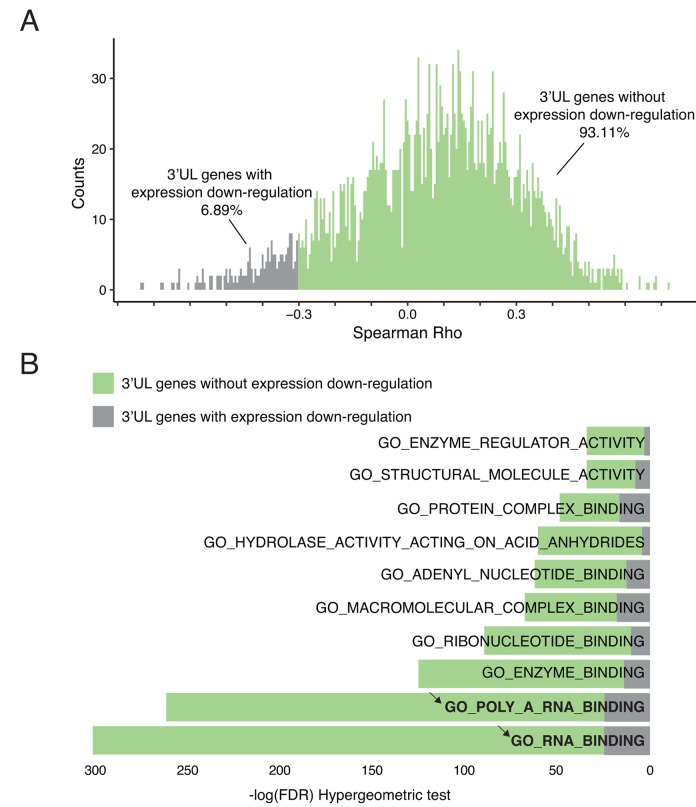
**3’UTR-longer genes do not affect their own expressions.** (**A**) 3’UL genes without direct downregulation expression predominate. Only 6.89% of 3’UL genes show direct down-regulated downstream expression. The downregulation was defined as Spearman Rho < -0.3 and P-value < 0.05. (**B**) 3’UL genes without down-regulated gene expression control more aspects of molecular functions (hypergeometric test, retrieved from Molecular Signature Database). RNA-binding ranks highest in the function enrichment computation.

3’UTR plays a critical role in the regulation of mRNA level. In general, short 3’UTR upregulates gene expression [23]. Longer 3’UTR may expose the RNA molecule in a more attackable manner. Therefore, we ask whether the 3’UTR lengthening after HPV infection will affect their own mRNA level. Among all 3’UL events, we compute the interplay between the APAome and transcriptome. Interestingly, our results suggest that only 6.89% of 3’UL genes shows direct down-regulated expression (Figure 2A). We also verify that differential APA do not fully explain the differential expression (Supplementary Figure 2A). It suggests that 3’UTR lengthening in HPV infected cells has minimal effect on their own gene expression. This is consistent with the previous report to show that only ~2% — 12% of APA events are negatively correlated with their gene expression [21].

3’UTR lengthening fails to influence their own gene expression, suggesting that these 3’UTR lengthening genes might not be the direct targets for HPV. This is supported by the following functional enrichment analysis. Based on the Molecular Signature Database, among the top 10 overlapped gene ontology enrichment terms, we do not observe any cancer-development-related terms (Figure 2B). In contrast, those genes are enriched in RNA binding, which indicates the potential regulatory roles of these 3’UL genes. In order to verify that 3’UL genes without down-regulation are not directly associated with biological functions, we examine whether they are involved in immune functions, tumor progression, and HPV infection. We apply six well-established molecular signatures and no significant results were observed (Supplementary Figure 2B). Taken together, our results suggest that 3’UL genes are unlikely the direct targets of HPV infection to transform cells. Instead, these 3’UL genes might regulate other genes by using their RNA binding affinity.

### Longer 3’UTR indirectly impacts genes by recruiting RNA-binding proteins

**Figure 3:**
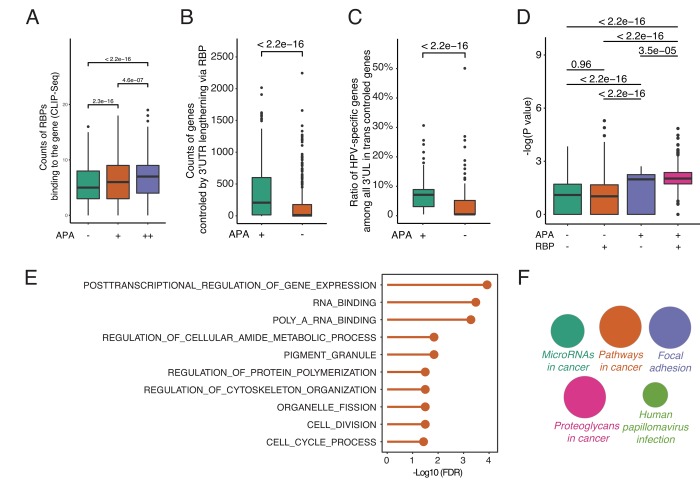
**By releasing intermediate RNA-binding proteins (RBPs), 3’UL genes indirectly impact paired genes in trans.** (**A**) Differential-APA genes are easier to be attacked by RBPs (Wilcoxon test, data from CLIP-seq). APA+ group and APA++ group represent different differential APA level (P < 0.05 and P < 0.001, respectively). (**B**) 3’UL controls more paired genes in trans compared with non-3’UL APA events. This computation was generated by randomly selecting 1,000 3’UL-gene pairs. In order to retrieve high-confidence interactions, we limit the number of intermediate RBPs > 3. (**C**) 3’UL-in-trans-paired genes show significantly higher ratio of HPV-specific. This computation was generated by randomly selecting 1,000 3’UL-gene pairs with the number of intermediate RBPs > 3. (**D**) Through intermediate RBPs, 3’UL controls paired genes in trans. APA+RBP+ group shows the most significant HPV-specific trend. The bigger the Y-axis value is, the group harbors more HPV-specific trend. The Y-axis marks the -log (P-value) of differential expression between HPV+ and HPV- samples through EdgeR. The P-value between 4 groups was computed through the Wilcoxon test. This computation was generated by randomly selecting 1,000 3’UL-gene pairs. RBP+/RBP- was defined as higher/lower RBP-binding confidence level (intermediate RBPs > 3 and ≤ 3, respectively). In the RBP+ group, 3’UL impacts in-trans-paired genes with higher confidence. (**E**) Biological process enrichment analysis of HUB-3’UL in-trans-paired genes (hypergeometric test, retrieved from Molecular Signature Database). The cell cycle is the most top ranking for enrichment term. HUB-3’UL was defined as the 3’UL genes with interacting RBPs ≥ 15. (**F**) Pathway enrichment of HUB-3’UL in-trans-paired genes. CLUEGO package was used based on KEGG database. The size of each bubble represents the enrichment level of each term.

To understand how 3’UL impacts downstream functions and the potential function of RBPs, we fetch the RBP-mRNA interactome data from RAID (July 2018) [24]. RAID is a comprehensive resource for evaluating the RNA-protein interaction based on CLIP-seq data. Under normal conditions, RBPs competitively bind to target RNAs and regulate their targets expression [25]. However, when genes with longer 3′UTRs are able to sequester more RBPs, the other targets of RBPs may get repressed or activated due to fewer interferences of RBPs. The analyses reveal that, in accordance with our hypothesis, differential APA genes are more capable of being bound by RBPs (Figure 3A). The degree of differential APA also tends to be correlated with the counts of RBPs binding to each gene. Next, we calculate the counts of paired genes controlled by 3’UL via RBPs. The paired genes are defined as the targets of 3’UTR associated RBPs (3’UTR-RBP-targets). Due to the large interactome dataset, the 3’UTR—RBP—gene pairs are randomly selected for 1000 times. Surprisingly, differential-APA genes profoundly impact their paired genes (P < 2.2e-16, Wilcoxon test, Figure 3B). In order to have a closer view of the paired genes, we compute the ratio of HPV-specific genes among them (randomly selected 1000 times). Consequently, the pairs of differential-APA genes are more enriched in HPV-specific molecules (Figure 3C). These data collectively suggest that the pairs of longer 3’UTR genes are enriched in HPV-specific genes. HPV regulates oncogenic genes by modifying the 3’UTR of a group of genes and subsequently affect the function of RBPs.

To further validate the APA—RBP—paired gene axis, we next explore whether the paired genes are in an RBP-dependent manner. We divide the interactome into two groups: RBP+ and RBP- groups, which are defined as higher or lower RBP-binding confidence level (intermediate RBPs > 3 and ≤ 3, respectively). The RBP+ group means APA regulates paired genes via more intermediate RBPs in a high confidence level. In contrast, RBP- group represents a weaker bridge between 3’UTR and paired genes. The Y-axis marks the -log (P-value) of differential expression between HPV+ and HPV- samples. The bigger the Y-axis value is, the group harbors a more HPV-specific trend. Firstly, among the APA negative groups, no significant difference of HPV-specific-trend is observed between RBP+ and RBP- sets (P = 0.96, Wilcoxon test). On the contrary, in the APA+RBP+ group, the -log (P-values) of differential expression are upregulated significantly compared with the other three groups (Figure 3D). This result strongly supports the hypothesis that APA regulates paired genes dependent on RBPs.

Next, to better quantify the functional impact of the global APA-changed paired genes, we monitor the associated biological processes. With a focus on the hub 3’UL genes, we limit the cutoff number of intermediate RBPs to ≥ 15. Interestingly, the cell cycle is the top ranking term, which is strongly in consistency with our previous understandings into HPV+ tumor [26,27] (Figure 3E). Further, pathway enrichment analysis was computed through CLUEGO. We set the most global network specificity to generate the most credible enrichment results. Strikingly, the hub-3’UL paired genes are strongly associated with HPV infection (Figure 3F). Thus, we provide convincing evidence to show that HPV shapes host cells by modifying the length of 3UTR, subsequently affect the function of RBPs and finally regulate their target genes for survival and transformation.

### 3’UTR lengthening of RBM25 upregulates PD-1 via FUS and DGCR8

**Figure 4:**
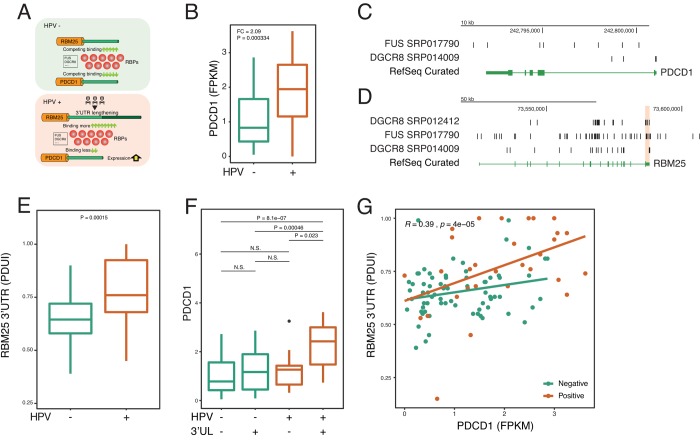
**HPV-mediated longer 3’UTR of RBM25 and GTF3C3 upregulates PD-1 via impacting RBPs of FUS and DGCR8.** (**A**) In the HPV+ state, 3’UL mediates the imbalance of competing for binding of RBPs. Longer 3’UTR of RBM25 and GTF3C3 drives more RBPs to bind on them. Conversely, less RBPs will bind on PDCD1, which upregulates the expression of PDCD1. (**B**) PDCD1 is upregulated in the HPV+ samples. Fold change and P-value were computed through EdgeR. (**C**) RBPs of FUS and DGCR8 can bind to the 3’UTR of PDCD1 (CLIP-seq). CLIP-seq peaks were combined within all samples. (**D**) RBPs of FUS and DGCR8 can bind to the 3’UTR of RBM25. CLIP-seq peaks were combined within all samples. (**E**) 3’UTR level of RBM25 is significantly longer in HPV+ samples (Wilcoxon test). (**F**) 3’UL+ and HPV+ group shows the highest PDCD1 expression (FPKM). (**G**) 3’UTR length of RBM25 is positively correlated with PDCD1 expression. Spearman Rho value and P value was analyzed in HPV positive and negative samples.

To further confirm the identified paradigm that HPV shapes host cells by using APA—RBP—paired gene axis, we next focus on individual key molecules to examine the paradigm (Figure 4A). A recent study reports HPV induces the dysfunction of CD8 T cells ^30^. Yet, very little is known about the underlying mechanism. Therefore, we asked whether HPV infection affects PD-1, which is a key inhibitory checkpoint of CD8 T cells. Interestingly, in accordance with previous reports [28], we confirm that HPV upregulates the expression of PD-1 (Figure 4B). Next, the upstream 3’UL genes were computed based on CLIP-seq. We rank the Spearman Rho between 3’UL genes and PD-1 and found that RBM25 and GTF3C3 are two potential regulators of PD-1 (Figure 4C). Next, we mine the interactome of RBPs and RNAs and observe two potential intermediate RBPs including FUS and DGCR8 (Figure 4C). In order to validate our computation based on experimental results, we use another independent CLIP-seq to confirm our findings. In consistency with the computational data, we confirm that FUS and DGCR8 bind to PD-1 and the RBM25 3’UTR by combining CLIP-seq and RNA-seq data (Figure 4C-4D).

To further determine whether HPV regulates PD-1 in the 3’UL dependent manner, we focus our analysis on the RNA-seq data. HPV infection can robustly increase the APA level of RBM25 and GTF3C3 (Figure 4E and Supplementary Figure 3A). Without 3’UL, HPV infection alone fails to significantly upregulate PD-1 expression (Figure 4F and Supplementary Figure 3B). In the presence of 3’UL, HPV infection dramatically increases the level of PD-1 (Figure 4F and Supplementary Figure 3B). Our analysis supports that HPV infection increases PD-1 expression by elongating the 3’UTR of RBM25 and GTF3C3. As expected, correlations between 3’UTR scores (PDUI) (RBM25 and GTF3C3) and PDCD1 expression (FPKM) also demonstrate the interplay between 3’UL and PDCD1 (Figure 4G). We next found that PABPN1, the core component of APA machinery, correlates with 3’UTR of RBM25 (Supplementary Figure 3D). We further examine whether HPV affects FUS or DRGR8 directly and find that HPV infection made insignificant changes to RNA expression of those two genes (Supplementary Figure 3E). Together, these results substantiate our hypotheses that 3’UTR lengthening contributes to upregulation of PD-1 via RBPs.

### 3’UTR lengthening upregulates cell cycle gene E2F1

To further prove our hypothesis that 3’UTR lengthening affects gene expression via RBP, we focus on the cell cycle process of cancer cells which is the hallmark of HPV infection [26,29]. Though lots of layers of this process were reported such as miRNAs and lncRNAs [30–32], the upper regulator is not fully understood. E2F1 is a key cell cycle regulator [33–35] and has long been regarded as an oncogene [35–38].

To further gain insights into associations between HPV and cell cycle regulator E2F1, we re-check the differential APA profile. Surprisingly, among the APAome, E2F1 undergo 3’UTR lengthening (Supplementary Figure 4A) indicating a higher likelihood of being downregulated by miRNAs and RBPs. In contrast with the 3’UTR lengthening, E2F1 is paradoxically upregulated in HPV+ samples (Fold change = 4.92, P = 1.45E-37; Supplementary Figure 4A). These opposite results suggest there exists another robust regulator of E2F1 that powerfully controls its expression. The APA—RBP—paired gene axis might be involved in the regulation of E2F1. Thus, after mining the interactome based on CLIP-seq data between RBPs and RNAs as is similarly conducted in Figure 4, we observe a regulatory network between E2F1 and 3’UL genes EDC3 and AGGF1 and demonstrate that interplay between 3’UL and E2F1 is RBP dependent (Supplementary Figure 4B). HPV infection could significantly upregulate the APA level of AGGF1 and EDC3 (Supplementary Figure 4C and Supplementary Figure 4F). Without 3’UL, HPV infection fails to upregulate E2F1 (Supplementary Figure 4D and Supplementary Figure 4G). However, in the presence of 3’UL, HPV significantly upregulates E3F1 (Supplementary Figure 4D and Supplementary Figure 4G). The APA score of AGGF1 and EDC3 highly correlates with the expression of E2F1 (Supplementary Figure 4E and 5H). Collectively, these results provide new evidence of how HPV alters cell cycles through changing 3’UTR.

## DISCUSSION

Viral infection greatly contributes to the global cancer burden, but how oncogenic virus transforms normal cells to cancerous cells is still poorly understood. Viral infection widely affects gene expression and several common virus-targeted pathways have been identified. For example, virus profoundly altered the miRNA profile and activate oncogenic processes [30]. However, we are convinced that those mechanisms may not be the whole picture. The molecular mechanism of how viral infection extensively influences gene expression is still not fully addressed. Here, we found a new routine which virus utilized to modify gene expression. In our analysis, HPV infection could significantly elongate 3’ UTR from many genes. These elongated 3’UTRs significantly affect the pool of RBP binding motif and thus inhibit RBP function. As a result, the other RBP binding targets were rescued and increased expression significantly. Through this mechanism, virus modifies 3’UTR to control the expression of a group of genes via the intermediate RBPs. Thus, our results might provide novel insights to understand how virus shape cell by modifying the length of 3’ UTRs. The model providing here might also be suitable for other virus and might be a useful paradigm to understand the interaction between virus and host.

The 3’UTRs of eukaryotic mRNA is generated through endonucleolytic cleavage at poly(A) site and subsequently polyadenylation by the poly(A) polymerases [39]. The choice of different poly(A) site determines the length of 3’UTRs and this process is orchestrated by a multi-protein machinery composed of three main complexes: 1) Cleavage and Polyadenylation Stimulatory Factor (CPSF), 2) Cleavage Stimulatory Factor (CSTF), and 3) Cleavage Factors Im and IIm (CFIm, CFIIm) [40,41]. After cleavage at poly(A) site, poly(A) tails are added by a poly(A) polymerase (PAP) and recruit poly(A)-binding proteins (PABPs) including the cytosolic PABPC and the nuclear PABPN1. PABPN1 directly interacts with PAS regions and suppresses the usage of weak proximal PASs [22]. In accordance with this mechanism, our data confirmed the loss of PABPN1 in human cells resulted in extensive 3’ UTR shortening. We observed significantly higher expression of PABPN1 in HPV infected cells. It suggests that HPV might regulate the key components such as PABPN1 in the 3′-end processing machinery to modify the length of 3’UTR.

Increasing evidence indicates that APA is extensively used and is an important way to regulate gene expression [42–44]. The 3’UTR influences many aspects of mRNA metabolism including but not limited to transcription termination by RNAP II, mRNA stability, mRNA export to the cytoplasm, and the efficiency of translation. The 3’ UTRs often harbor regulatory elements such as AU-rich elements (AREs) or other binding motifs that are easily attacked by micro-RNA (miRNA) [45,46]. Previous studies show that transcripts with shorter 3’ UTRs are more likely to produce higher levels of protein [47,48]. Interestingly, in our analysis, the status of 3’UTRs do not affect their own RNA level. Instead, they significantly influence their binding RBP’s downstream targets. However, it is still possible that the changed 3’UTRs might affect their protein translation even though they not affect mRNA level directly. While this possibility was not directly excluded due to lack of corresponding sequencing data at the protein level. But this possibility was not supported by alternative approaches. For example, functional annotation of RBP’s downstream targets but not 3’UTR resident genes was enriched in the cell cycle, which is highly related to virus infection and replication. Therefore, our finding provides a new insight to reveal how virus shapes host transcriptional profile for their survival and replication.

Pan-cancer analysis across 7 cancers including 358 tumor/normal pairs reveals that most APA genes (91%) in tumors have shorter 3′ UTRs. Besides these global features of APA events in cancer, several specific APA regulated genes in different cancer have also been investigated. For example, in hepatocellular carcinoma, downregulation of CFIm25 subunit of cleavage factor results in high expression of PSMB2 and CXXC5 by increasing the usage of the proximal polyadenylation site [49]. In pancreatic ductal adenocarcinoma, the increased ZEB1 protein expression is due to its shorted 3’UTR [50]. In breast cancer, the cellular proliferation marker Ki-67 is increased due to shortening of the Ki-67 3’UTR [51]. To our surprise, HPV infection is significantly associated with elongated 3’UTR. It suggests that the HPV-induced cancer might use a different mechanism to drive cancer. It remains an interesting question to further test the difference between virus-induced cancers and mutation-driven cancer. This is critically important for us to prevent and treat cancer based on their driving factors.

In a nutshell, by comparing the APA events between HPV infected or non-infected cancers, we found HPV-induced features of APA. Through elongating the 3’UTRs, HPV indirectly changes the RBP targets for their survival and cell transformation. Our work not only presents a novel paradigm of how oncogenic viruses shape tumor transcriptome by modifying the 3’UTR, but also highlight a previously unrecognized layer of APA—RBP interplay in this molecular hierarchy. Modification of the pool of RBP-binding motif might provide new insight to understand virus-associated carcinogenesis.

## MATERIALS AND METHODS

### Data sets and data availability

All the RNA-seq data were fetched from The Cancer Genome Atlas. We retrieved the level 3 transcriptome raw counts and FPKM profile of head and neck squamous carcinoma samples. The HPV positive/negative statuses were defined by the p16 testing results. Samples without HPV status and their RNA-seq data were excluded for computation. Non-tumor samples were excluded. In all, 111 patient samples were included in the analysis, including 73 HPV negative samples and 38 HPV positive samples. The detailed patient clinical information can be seen in Supplementary Table 1.

### Alternative polyadenylation events quantification

The Cancer 3’UTR Atlas yields the characterization of APAome based on RNA-seq by using DaPars [16,52] (https://github.com/ZhengXia/DaPars). APAome refers to all APA events detected by DaPars. To infer the proximal polyadenylation sites, 3’UTR annotation was generated based on UCSC hg19 reference (python DaPars_Extract_Anno.py -b gene.bed.txt -s symbol.txt -o 3UTR.bed). The Percentage of Distal polyA site Usage Indexes (PDUIs) was computed for all transcripts (python DaPars_main.py my_configure_file.txt). The PDUI score ranges from 0 to 1, which indicate from full shortening to full lengthening of 3’UTR. The larger PDUI indicates the longer 3’UTR that each transcript has. We used the Wilcoxon test for differential APA analysis which has been used before [21]. APA events without enough PDUI scores were excluded for analysis. We define P < 0.05 as a significant differential APA event. We use pheatmap package for visualizing the heat map of APA profiles.

### Cross-linking immunoprecipitation sequencing analysis

In order to assess the impact of 3’UTR changing, we fetched the RBP-RNA interactome data from RAID [24] (July 2018). In order to retrieve high-confidence interactions between 3’UL genes and paired genes, we regard the pairs with the number of intermediate RBPs > 3 as high-confidence interactions. HUB-3’UL was defined as the 3’UL genes with interacting RBPs > 15. StarBase was used to search the interacting loci between RBP and transcript [53]. The cross-linking immunoprecipitation sequencing (CLIP-seq) data was fetched from NCBI Sequence Read Archive (accession number SRP014009, SRP017790, and SRP012412).

### RNA sequencing analysis

The hg19 genome version was used for transcript annotation. Differential expression analysis was computed through EdgeR with the raw counts of all transcripts as input. We set the cutoff value of P < 0.001 and |fold change| > 2. In the correlation analysis, we define the statistical significance with the |Spearman Rho| > 0.3 and P-value < 0.05.

### Functional enrichment annotation

Gene ontology enrichment and pathway enrichment annotation were computed through Molecular Signature Database (http://software.broadinstitute.org/gsea/msigdb/index.jsp) and CLUEGO.

### Survival analysis and principle component analysis

Patients were split according to the viral status and the longest survival time was set as two years. The P-value for survival analysis was computed through the log-rank test. Principle component analysis was analyzed based on ggfortify package in R. APA events with null values were discarded for principal component analysis urged by ggfortify package.

## Supplementary Material

Supplementary Figures

Supplementary Table
