# Supplementary material for: HPV shapes tumor transcriptome by globally modifying the pool of RNA binding protein-binding motif
Source: Aging (Albany NY). 2019 Apr 29;11(8):2430–46. doi: 10.18632/aging.101927 (PMC6520004; doi:10.18632/aging.101927)
Supplement: Supplementary Figures [file aging-11-101927-s001.pdf]

SUPPLEMENTARY TABLE

Supplementary Table 1. The clinical information of patients included in the analysis.

			HPV positive (n = 38)		HPV negative (n = 73)
**Age**					
	Median (range)		56 (40-71)		60 (19-83)
**Stage**					
	I	2	5.3%	4	5.5%
	II	6	15.8%	9	12.3%
	III	5	13.2%	13	17.8%
	IV	25	65.8%	46	63.0%
	Unknown	0	0.0%	1	1.4%
**Gender**					
	Male	35	92.1%	56	76.7%
	Female	3	7.9%	17	23.3%
**Smoking history**					
	Never	11	28.9%	11	15.1%
	Former	11	28.9%	31	42.5%
	Current	15	39.5%	30	41.1%
	Unknown	1	2.6%	1	1.4%
**Alcohol history**					
	YES	29	76.3%	54	74.0%
	NO	9	23.7%	17	23.3%
	unknown	0	0.0%	2	2.7%
