# Supplementary material for: HPV shapes tumor transcriptome by globally modifying the pool of RNA binding protein-binding motif
Source: Aging (Albany NY). 2019 Apr 29;11(8):2430–46. doi: 10.18632/aging.101927 (PMC6520004; doi:10.18632/aging.101927)
Supplement: Supplementary Table [file aging-11-101927-s002.pdf]

SUPPLEMENTARY FIGURES

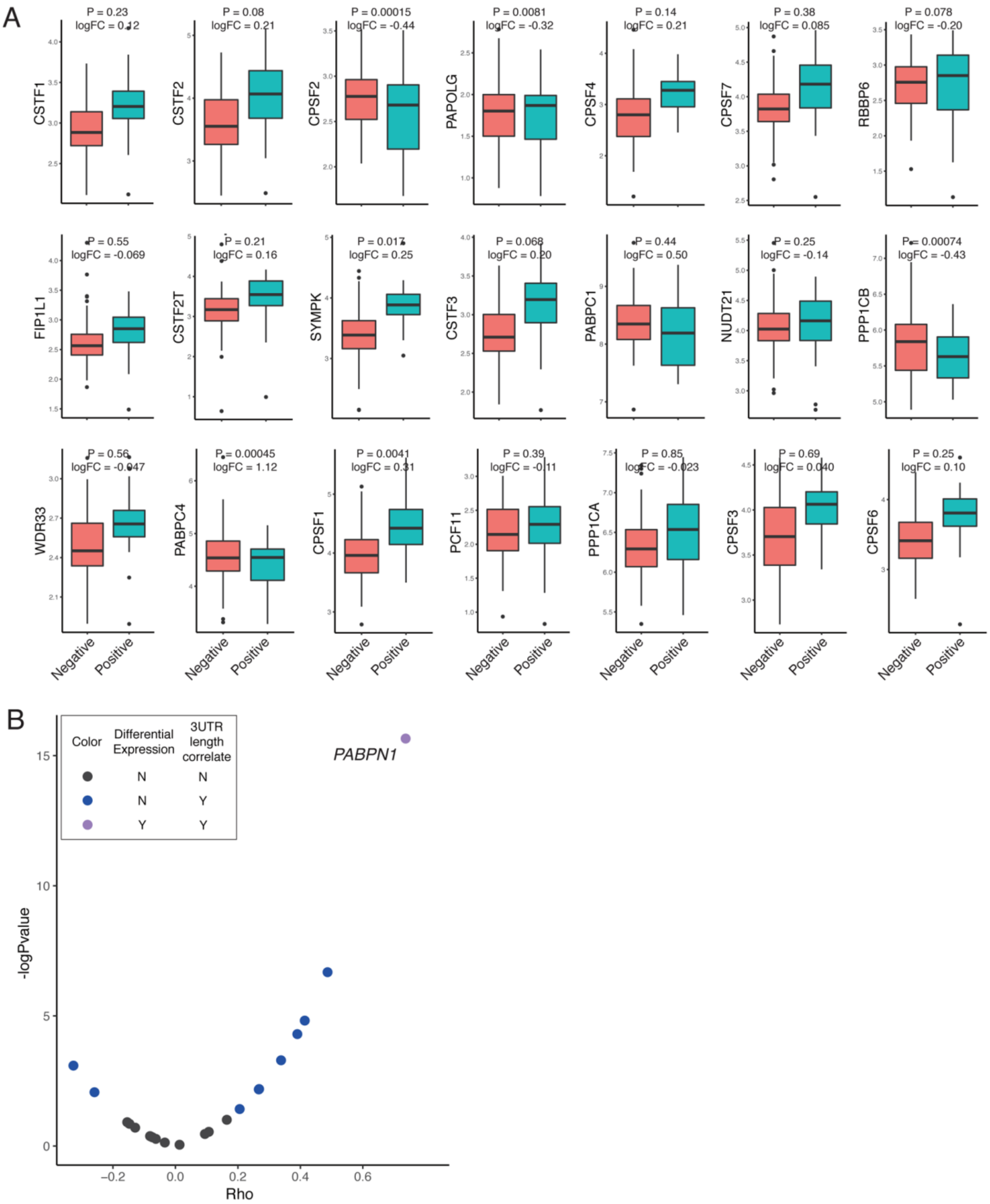

Supplementary Figure 1. The expression profile of key APA regulation components. (**A**) The list of regulators was fetched from the literature (Ref. 20). The Y-axis refers to the FPKM value of each mRNA. (**B**) Scatter plot of all components shows PABPN1 plays key roles in HPV-mediated APA. Each dot represents each known APA regulators, colored according to whether this regulator is differentially expressed in HPV+ samples or whether the expression is correlated with the median 3'UTR length of all APA events (|Spearman Rho| > 0.3 and P-value < 0.05).

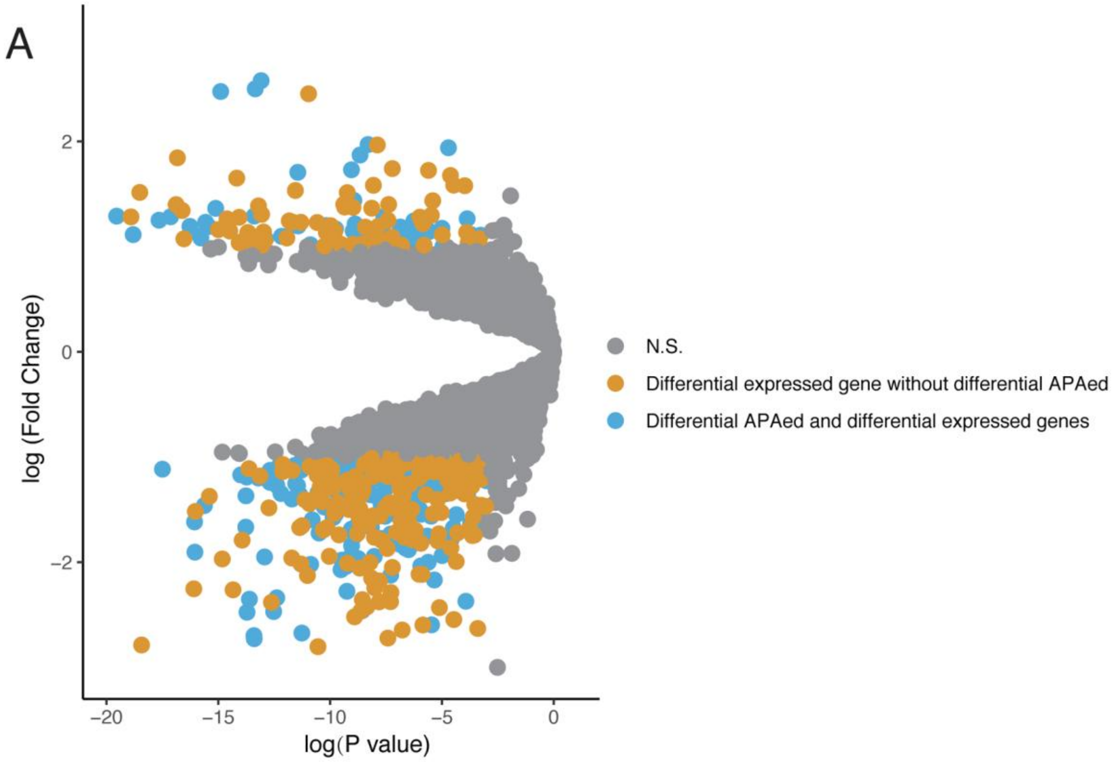

B

Gene sets	P value (hypergeometric test)	Significance
**Tumor supressor (Uniprot)**	0.4992319	N.S.
**Oncogene (Uniprot)**	0.7815282	N.S.
**Tumor supressor (TSGD)**	0.9227407	N.S.
**Oncogene (NCG)**	0.998491	N.S.
**Immune genes (Bindea 2013)**	0.7493049	N.S.
**HPV changed genes (MSigDB)**	0.7229499	N.S.

Supplementary Figure 2. 3'UTR-longer genes cannot explain the differential expression and are not enriched in cancer-associated pathways. (**A**) Differentially APA genes are not enriched in differentially expressed genes. (**B**) 3'UL genes with direct down-regulated gene expression were not associated with cancer and immunity. All 6 signature shows no significant results (hypergeometric test).

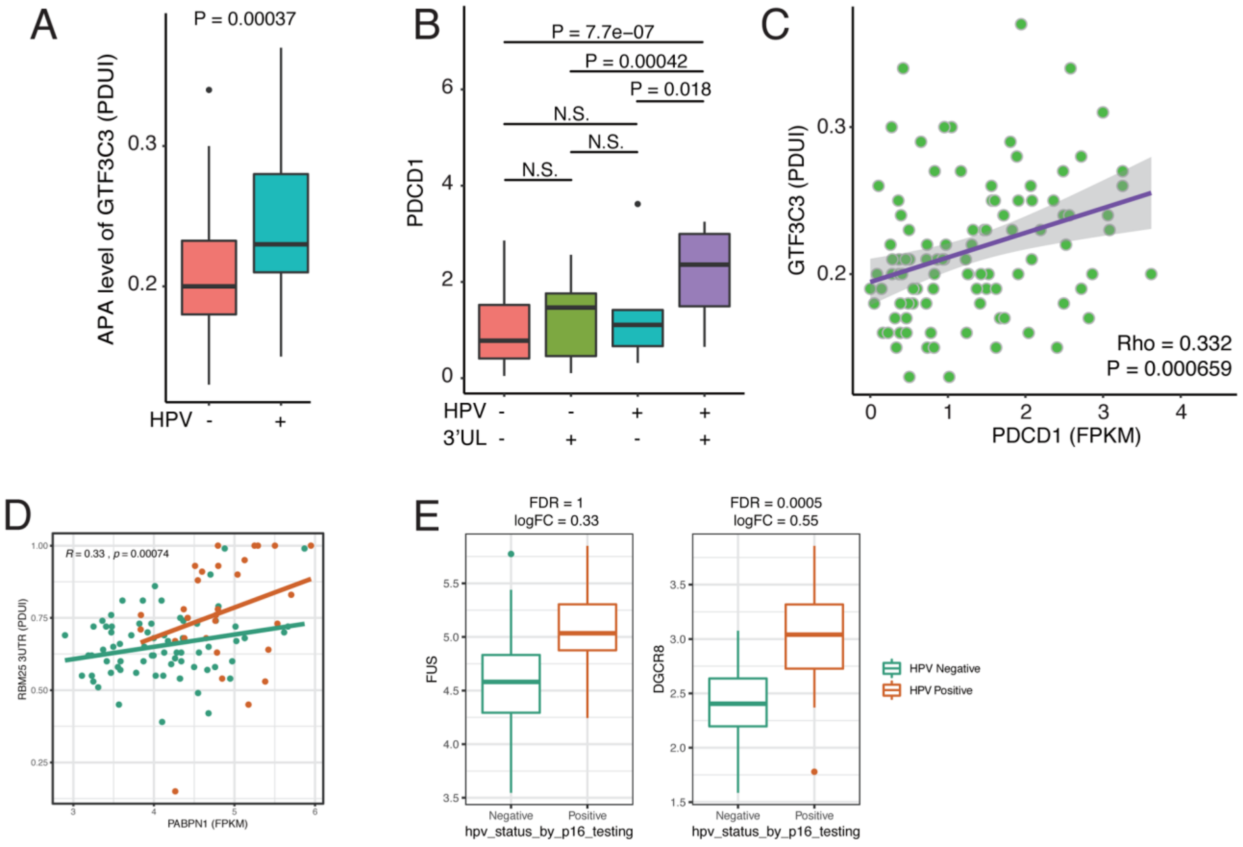

Supplementary Figure 3. HPV upregulates PD-1 via impacting RBPs. (**A**) 3'UTR level of GTF3C3 is significantly longer in HPV+ samples (Wilcoxon test). (**B**) 3'UL+ and HPV+ group shows the highest PDCD1 expression (FPKM). (**C**) 3'UTR length of GTF3C3 is positively correlated with PDCD1 expression. (**D**) Correlation between 3'UTR length of RBM25 and PABPN1 RNA expression. (**E**) The expression difference of FUS and DGCR8 between HPV positive and negative samples.

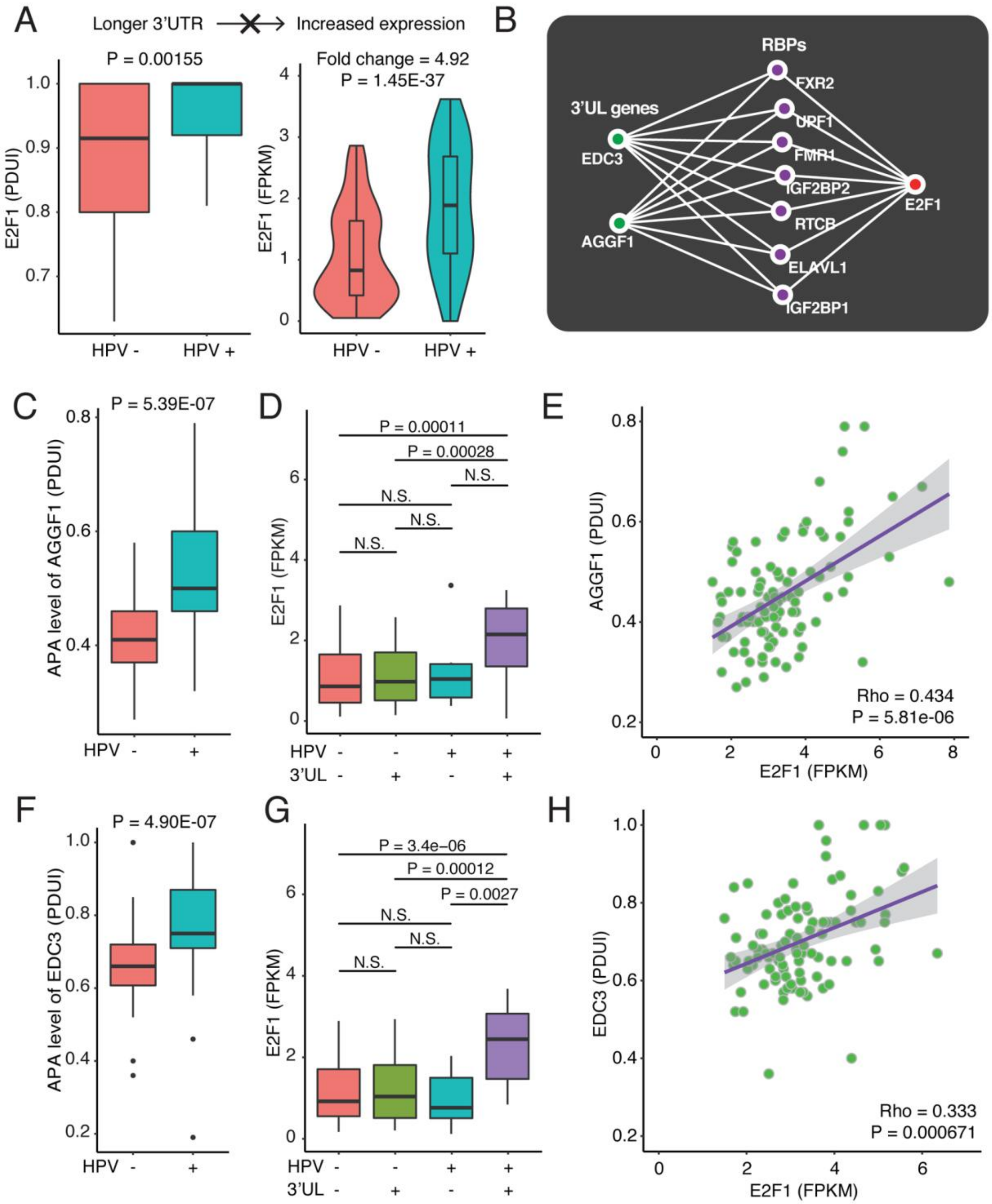

Supplementary Figure 4. HPV-mediated longer 3'UTR of AGGF1, EDC3 upregulates E2F1 via RBPs. (**A**) HPV+ samples show a longer 3'UTR length of E2F1. However, E2F1 is upregulated in HPV+ samples. The down-regulated expression of E2F1 may not be caused by 3'UL in cis. The right plot means PDCD1 is upregulated in the HPV+ samples. Fold change and P-value were computed through EdgeR. The right plot means. (**B**) Based on CLIP-seq, the 3'UL—RBP—E2F1 network was computed. (**C**) 3'UTR level of AGGF1 is significantly longer in HPV+ samples (Wilcoxon test). (**D**) 3'UL+ and HPV+ group shows the highest E2F1 expression (FPKM). (**E**) 3'UTR length of AGGF1 is positively correlated with E2F1 expression. (**F**) 3'UTR level of EDC3 is significantly longer in HPV+ samples (Wilcoxon test). (**G**) 3'UL+ and HPV+ group shows the highest E2F1 expression (FPKM). (H) 3'UTR length of EDC3 is positively correlated with E2F1 expression.
